# An other-race effect for configural and featural processing of faces: upper and lower face regions play different roles

**DOI:** 10.3389/fpsyg.2015.00559

**Published:** 2015-05-08

**Authors:** Zhe Wang, Paul C. Quinn, James W. Tanaka, Xiaoyang Yu, Yu-Hao P. Sun, Jiangang Liu, Olivier Pascalis, Liezhong Ge, Kang Lee

**Affiliations:** ^1^Department of Psychology, Zhejiang Sci-Tech University, HangzhouChina; ^2^Department of Psychological and Brain Sciences, University of Delaware, Newark, DEUSA; ^3^Department of Psychology, University of Victoria, Vancouver, BCCanada; ^4^Dr. Eric Jackman Institute of Child Study, University of TorontoToronto, ON, Canada; ^5^School of Computer and Information Technology, Beijing Jiaotong University, BeijingChina; ^6^Laboratoire de Psychologie et Neurocognition, Université Grenoble Alpes, ParisFrance

**Keywords:** face processing, face recognition, other-race effect, Face Dimensions Test, configural information, featural information, upper vs. lower face

## Abstract

We examined whether Asian individuals would show differential sensitivity to configural vs. featural changes to own- and other-race faces and whether such sensitivity would depend on whether the changes occurred in the upper vs. lower regions of the faces. We systematically varied the size of key facial features (eyes and mouth) of own-race Asian faces and other-race Caucasian faces, and the configuration (spacing) between the eyes and between the nose and mouth of the two types of faces. Results revealed that the other-race effect (ORE) is more pronounced when featural and configural spacing changes are in the upper region than in the lower region of the face. These findings reveal that information from the upper vs. lower region of the face contributes differentially to the ORE in face processing, and that processing of face race is influenced more by information location (i.e., upper vs. lower) than by information type (i.e., configural vs. featural).

## Introduction

Observers are generally better at recognizing and discriminating own-race faces relative to other-race faces (see Meissner and Brigham, 2001; Anzures et al., 2013, for a review). This phenomenon is called the other-race effect (ORE). In a standard recognition procedure, participants have to discriminate target faces learned in a study phase from distractor faces shown in a test phase. The ORE is reflected by a crossover interaction in discrimination accuracy or response time between the race of participants and the race of stimulus faces (Chance and Goldstein, 1987; Valentine, 1991; Michel et al., 2006b).

A holistic face processing view has been proposed to account for the ORE. This view begins with the idea that a face “schema” forms a holistic representation that selectively integrates face information into a perceived whole (Galton, 1879; Goldstein and Chance, 1980; Sergent, 1984; Tanaka and Farah, 1993). It has been claimed that own-race faces are processed more holistically than other-race faces (see Rossion and Michel, 2011, for a review). The holistic account of the ORE has been assessed using the established measures of holistic processing (Tanaka and Gordon, 2011): the parts/wholes task, the face composite task, and face inversion task. Using the part-whole paradigm (Tanaka and Farah, 1993), Tanaka et al. (2004) reported that Caucasian individuals from a mono-racial background showed a greater recognition benefit when Caucasian face parts were tested in a whole face context than when tested in isolation relative to Asian face parts. Michel et al. (2006a) found that both Belgian and Asian participants showed an ORE in an old/new recognition task and a greater interference for own-race than other-race faces in the composite-face task. Finally, Hancock and Rhodes (2008) found that both Caucasian and Asian participants showed that inversion more severely disrupted the recognition of own-race faces than other-race faces. These findings taken together suggest that holistic information plays an important role in the ORE.

Now, what additional facial information is crucial for the ORE? Traditionally, configural information (the metric relations that separate features) as opposed to featural information (the shape and size of the eyes, nose, and mouth) has been thought to play the predominant role in face processing in general and the ORE of face processing in particular. For example, it has been hypothesized that inversion (Yin, 1969) disproportionately disrupts the process of configural information (Freire et al., 2000; see Maurer et al., 2002, for a review). However, subsequent studies have shown that featural information does not necessarily play a lesser role than configural information in face processing in general (e.g., Taschereau-Dumouchel et al., 2010; Tanaka et al., 2014a) and the ORE in particular. With regard to the ORE, Rhodes et al. (2006) used a sequential matching task and found that Caucasian and Asian participants showed better recognition of both individual features and their spatial relations for upright own-race faces than for upright other-race faces. Likewise, Hayward et al. (2008) and Rhodes et al. (2009) used blurred and scrambled faces in a recognition task to dissociate and manipulate the structure and component information in a face and found that participants performed better for own-race than other-race faces for both the blurred wholes and scrambled components. These findings suggest that the ORE is sensitive to both configural and featural information in a face, rather than selectively relying on only one type of processing.

However, because faces are perceived on multiple featural and spatial dimensions, it is unlikely that own-race faces are processed in a superior way than other-race faces on every dimension. One relevant dimension is face location, with multiple lines of evidence suggesting that the upper vs. lower regions of a face are processed differentially. For example, “top-heavy” face-like patterns have been shown to direct preferential looking in newborn infants (Turati et al., 2002). In addition, sensitivity to information around the eyes begins and matures earlier than sensitivity to information around the nose and mouth in infants (Taylor et al., 2001; Liu et al., 2013). Similarly, eyes are better recognized than the nose or mouth in children’s face recognition and discrimination (Goldstein and Mackenberg, 1966; Hay and Cox, 2000; Pellicano and Rhodes, 2003; Pellicano et al., 2006; Ge et al., 2008; Tanaka et al., 2014b). Moreover, eye features are more heavily utilized than nose or mouth features in face recognition by adults (Walker-Smith, 1978; Sergent, 1984; Tanaka and Farah, 1993), and the eye region has been shown to be the most diagnostic region for face identification in a gaze-tracking study (Peterson and Eckstein, 2012) and in image-based computational analyses (Sekuler et al., 2004; Vinette et al., 2004; Keil, 2008). Functional neuroimaging studies with adults have further shown that the right FFA is tuned to process curvilinear symmetrical patterns with high-contrast elements in the upper region (Caldara et al., 2006; Caldara and Seghier, 2009). Clinically, in individuals with autism (Wolf et al., 2008) or prosopagnosia (Caldara et al., 2005; Bukach et al., 2008; Rossion et al., 2009) who show specific face recognition deficits, the ability to detect change in the mouth region is preserved, but in the eye region is impaired.

To measure sensitivity to configural and featural changes in own- and other-race faces, we used the Face Dimensions Test designed by Bukach et al. (2008) and Tanaka et al. (2009). This test has been used previously to document face processing performance with behavioral and eye tracking measures in healthy children and adults (Xu and Tanaka, 2013; Tanaka et al., 2014a,b), individuals with autism (Wolf et al., 2008), prosopagnosic patients (Bukach et al., 2008; Rossion et al., 2009), and infants (Quinn and Tanaka, 2009; Quinn et al., 2013).

In the current study, participants were asked to detect changes in own- and other-race faces that differed in: (1) eye spacing (distance between the eyes), (2) mouth spacing (distance between nose and mouth), (3) eye feature (size of the eyes), or (4) mouth feature (size of the mouth), at easy, moderate, and difficult discrimination levels (Tanaka and Gordon, 2011). Half of the faces were own-race faces and half were other-race. In this way, we aimed to determine how the configural and featural aspects of the upper and lower regions contribute to the ORE. In particular, we sought to answer the following questions: (1) What types of information contribute to the ORE: configural (spacing), featural (size), or both? and (2) Which regions in the face give rise to an ORE: upper, lower, or both?

Expectations for performance can be derived from three different views of face processing. By the configural view (i.e., configural information in a face plays the predominant role in generating the ORE), participants should show an ORE in conditions where the spacing information between the two eyes or between the nose and mouth are manipulated, but not in conditions where the size information of the eyes or mouth is manipulated. Alternatively, by the configural + featural view (i.e., configural and featural information are equally important to the ORE), participants should show an ORE in both the spacing and size conditions, regardless of whether information is manipulated in the upper or lower regions. Finally, if the upper and lower regions of a face make different contributions to the ORE (i.e., changes in the upper region are more important than changes in the lower region in producing the ORE), participants should show significantly more of an ORE, for both spacing and size changes, in the upper face region than in the lower face region.

## Experiment 1: Detection of Spacing Differences

### Method

#### Participants

Nineteen Chinese college students (seven females) served as participants (Mean age = 21.1 years, SD = 2.2, age range: 18–25), with normal or corrected-to-normal vision. The students had no direct contact with any foreign individuals. The Institutional Review Board of Zhejiang Sci-Tech U approved the experiment.

#### Materials

Eight Asian and eight Caucasian male face photos were selected by a typicality-rating task (with a 7-point Likert scale) out of 36 Asian and 36 Caucasian male faces. The mean (and SD) typicality ratings of the selected eight Asian and Caucasian faces were 5.29 (0.17) and 5.17 (0.19), respectively. There was no jewelry, glasses, or makeup on these faces. Facial markings, if any, were removed. We transformed these photographs into gray-scale faces (approximately 250 pixels in width and 320 pixels in height). The visual angle of one face on the PC screen to an observer was approximately 7.9°× 9.5°.

**FIGURE 1:**
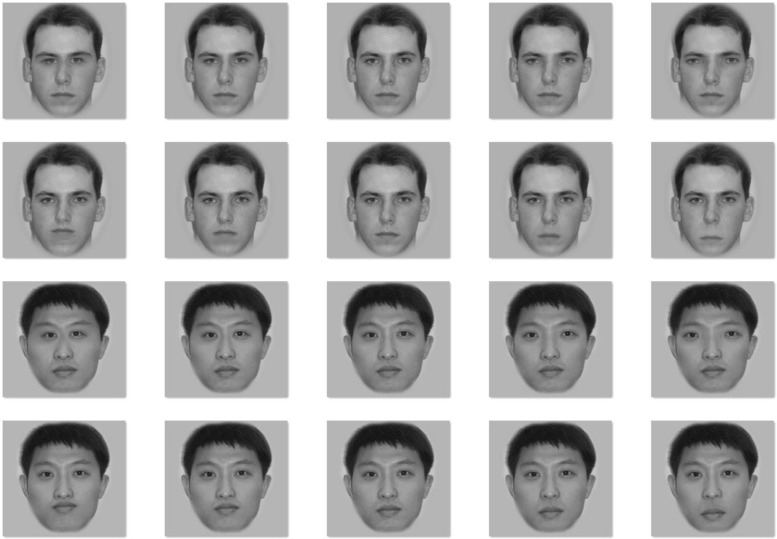
**Examples of spacing change between the two eyes (Rows 1 and 3) or between the nose and mouth (Rows 2 and 4) in a Caucasian face (Rows 1 and 2) and an Asian face (Rows 3 and 4)**. The middle face of each row is the original face upon which the manipulations were made. The leftmost face has the distance between eyes or between nose and mouth 10 pixels smaller than the original (the middle face), the face located second from the left has the distance between eyes or between nose and mouth 5 pixels smaller than the original, the rightmost face has the distance between eyes or between nose and mouth 10 pixels larger than the original, and the face located second from the right has the distance between eyes or between nose and mouth 5 pixels larger than the original.

The mean distances (and SDs) between the eyes of the original Caucasian and Asian faces were 85 (3.4) and 87 (2.1) pixels, respectively, without significant difference between the two races, *t*(14) = 1.59, *p* = 0.133. The mean distances (and SDs) between the nose and mouth of the original Caucasian and Asian faces were 47 (4.0) pixels and 49 (5.0) pixels, respectively, without significant difference between the two races, *t*(14) = 0.88, *p* = 0.393. Using the same manipulations as those in the Face Dimensions Test, we created eight different versions of each face by changing the amount of spacing between the eyes in the upper region (i.e., moving the two eyes 5 or 10 pixels closer or farther away from each other) and between the nose and mouth in the lower region (i.e., moving the mouth closer or farther away by 5 or 10 pixels from the nose) of the face. **Figure 1** shows example variants for an Asian and Caucasian male face.

#### Procedure and Design

Participants were asked to judge whether the two faces were the ‘same’ or ‘different’ with the instruction that a ‘same’ response indicates that the faces were judged to be physically identical. The same pairs presented two identical faces. The different pairs included three levels of difficulty in detecting the change: Easy, Medium, and Hard, with a 15-, 10-, or 5-pixel spacing difference, respectively, in the upper region (between the two eyes) or in the lower region (between the nose and mouth) of the two faces. On each trial, following a 150 ms fixation cross located in the screen center, a pair of photos was presented, side-by-side, with a time limit of 3,000 ms or until the participant pressed one of two keys responding ‘same’ or ‘different’, and this was followed by a 200 ms blank screen.

A full 2 (Race: Own or Other) × 2 (Region: Upper or Lower) × 3 (Level: Easy, Medium, or Hard) within-subject design was used. For each of the three levels of “different” manipulations in both the upper and lower regions, there were 40 trials presented (the 10 Asian and 10 Caucasian faces were both used twice). In total, 240 “different” pairs (as experimental trials) and 240 “same” pairs (as control trials) were mixed and presented randomly.

### Results and Discussion

**FIGURE 2:**
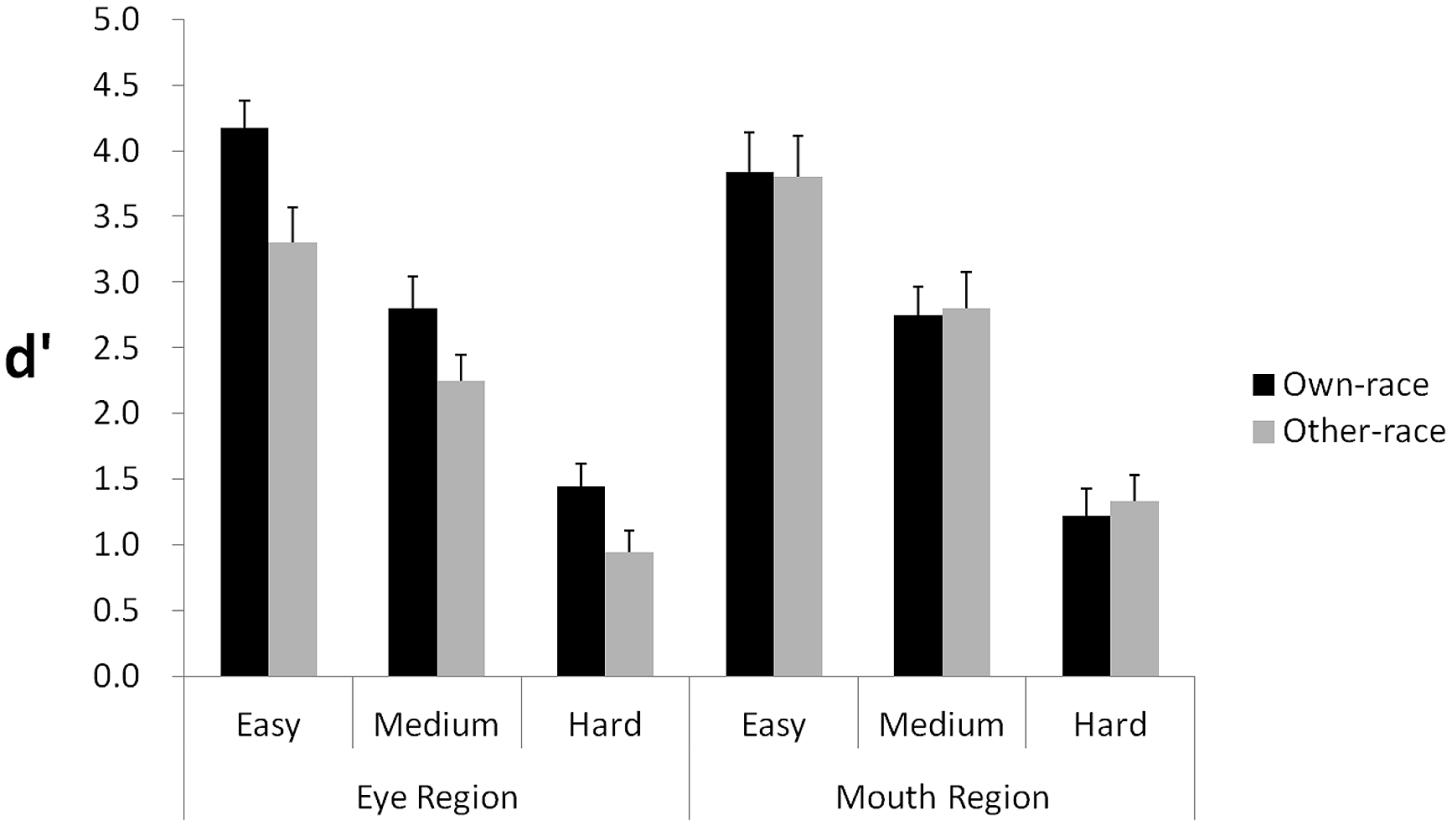
**Experiment 1: manipulating the spacing distance of facial features**. The graph shows *d*′ scores for detecting spacing differences across three levels of change (easy, medium, hard) in the eye and mouth regions, respectively. Error bars represent SEs in each condition.

By using correct “different” responses as Hits, incorrect “different” responses as False Alarms, the equation H = P(“different”—Different) = Φ[(-k + d′)/$\sqrt{2}$] + Φ[(-k - d′)/$\sqrt{2}$], and the equation FA = P(“different”—Same) = 2Φ(-k/$\sqrt{2}$), *d*′ scores were calculated as the dependent measure (Macmillan and Creelman, 2005, p. 197). Results are shown in **Figure 2**.

A 2 (Race: own, other) × 2 (Region: upper, lower) × 3 (Level: Easy, Medium, Hard) repeated measures analysis of variance was performed, with all three independent variables as within-subjects factors. The main effect of Race was significant, *F*(1,18) = 8.16, *p* = 0.010, $\eta_{p}^{2}$ = 0.312, demonstrating that discrimination performance for own-race faces was superior to discrimination performance in other-race faces. The main effect of Level was also significant, *F*(2,36) = 192.98, *p* < 0.001, $\eta_{p}^{2}$ = 0.915, indicating that performance varied according to the three levels of discrimination. The main effect of Region was not significant, *F*(1,18) = 0.39, *p* = 0.541, showing that discriminations in the upper face region did not differ from discriminations in the lower face region. Crucially, we found a significant interaction between Race and Region, *F*(1,18) = 9.33, *p* = 0.007, $\eta_{p}^{2}$ = 0.341. No other two-way interactions were significant. The three-way interaction of Race, Region, and Level was not significant, *F*(2,36) = 0.20, *p* = 0.823.

To explore the significant two-way interaction between Race and Region, we performed ANOVAs on the data for the upper and lower regions separately. For the upper region, a 2 (Race: Own vs. Other) × 3 (Level: Easy, Medium, Hard) repeated-measures ANOVA was performed. The main effect of Race was significant, *F*(1,18) = 32.24, *p* < 0.001, $\eta_{p}^{2}$ = 0.642, indicating that participant response accuracy was higher for own-race faces than for other-race faces, thereby providing evidence of an ORE in the upper region. The main effect of Level was significant, *F*(2,36) = 108.48, *p* < 0.001, $\eta_{p}^{2}$ = 0.858, indicating that the more spacing difference between two faces, the better participants differentiated them. The interaction of Race and Level was not significant, *F*(2,36) = 1.25, *p* > 0.29, indicating that the magnitude of the ORE in the upper region of the face does not dramatically change with changes in the magnitude of the spacing difference.

For the lower region, a 2 (Race: Own vs. Other) × 3 (Level: Easy, Medium, Hard) repeated-measures ANOVA was performed. The main effect of Race was not significant, *F*(1,18) = 0.053, *p* > 0.82, indicating that participant response accuracy was not different for own- versus other-race faces, thereby providing no evidence of an ORE. However, the main effect of Level was significant, *F*(2,36) = 138.44, *p* < 0.001, $\eta_{p}^{2}$ = 0.885, indicating that the more spacing difference between two faces, the better participants differentiated them. The interaction of Race and Level was not significant, *F*(2,36) = 0.096, *p* > 0.90.

Collectively, the findings indicate that the ORE in response accuracy was manifested in sensitivity to spacing change in the upper region; however, spacing difference in the lower region did not produce an ORE.

## Experiment 2: Detection of Feature Size Differences

Experiment 1 showed that spacing change in the upper face, but not in the lower face, influenced the ORE. However, it is still unclear whether this location effect would also be observed for featural (i.e., size) changes in the face. Experiment 2 examined this issue by manipulating eye and mouth size on multiple levels and comparing participant performance for detecting the difference between own-race faces and other-race faces.

### Method

#### Participants

Nineteen Chinese college students (14 females) served as participants (Mean age = 20.32 years, SD = 1.87, age range: 18–24), with normal or corrected-to-normal vision. The students had no direct contact with any foreign individuals. The Institutional Review Board of Zhejiang Sci-Tech U approved the experiment.

#### Materials

**FIGURE 3:**
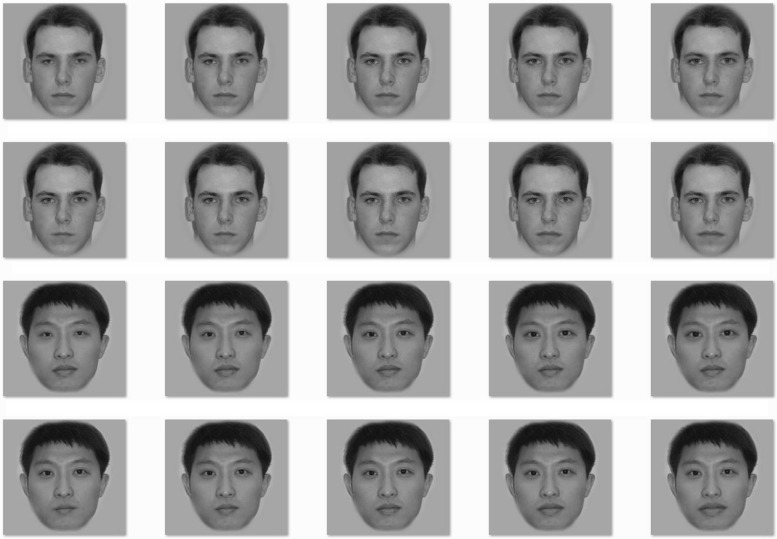
**Examples of size change of the two eyes (Rows 1 and 3) or the mouth (Rows 2 and 4) in a Caucasian face (Rows 1 and 2) and an Asian face (Rows 3 and 4)**. The middle face of each row is the original face upon which the manipulations were made. The leftmost face has eyes or mouth 20% smaller than the original (the middle face), the face located second from the left has eyes or mouth 10% smaller than the original, the rightmost face has eyes or mouth 20% larger than the original, and the face located second from the right has eyes or mouth 10% larger than the original.

Stimuli were created in the same fashion as in Experiment 1. However, the manipulation was of size instead of spacing (i.e., increasing or decreasing the size of the eyes or mouth by 10 or 20%). The means (and SDs) of eye size (width) of the original Caucasian and Asian faces were 39 (3.2) and 38 (2.8) pixels, respectively, without significant difference between the two races, *t*(14) = 1.17, *p* = 0.262. The means (and SDs) of mouth size (width) of the original Caucasian and Asian faces were 69 (6.9) pixels and 70 (6.6) pixels, respectively, without significant difference between the two races, *t*(14) = –0.30, *p* = 0.771. **Figure 3** shows example variants for an Asian and Caucasian male face.

#### Procedure and Design

The procedure and design were the same as in Experiment 1.

### Results and Discussion

**FIGURE 4:**
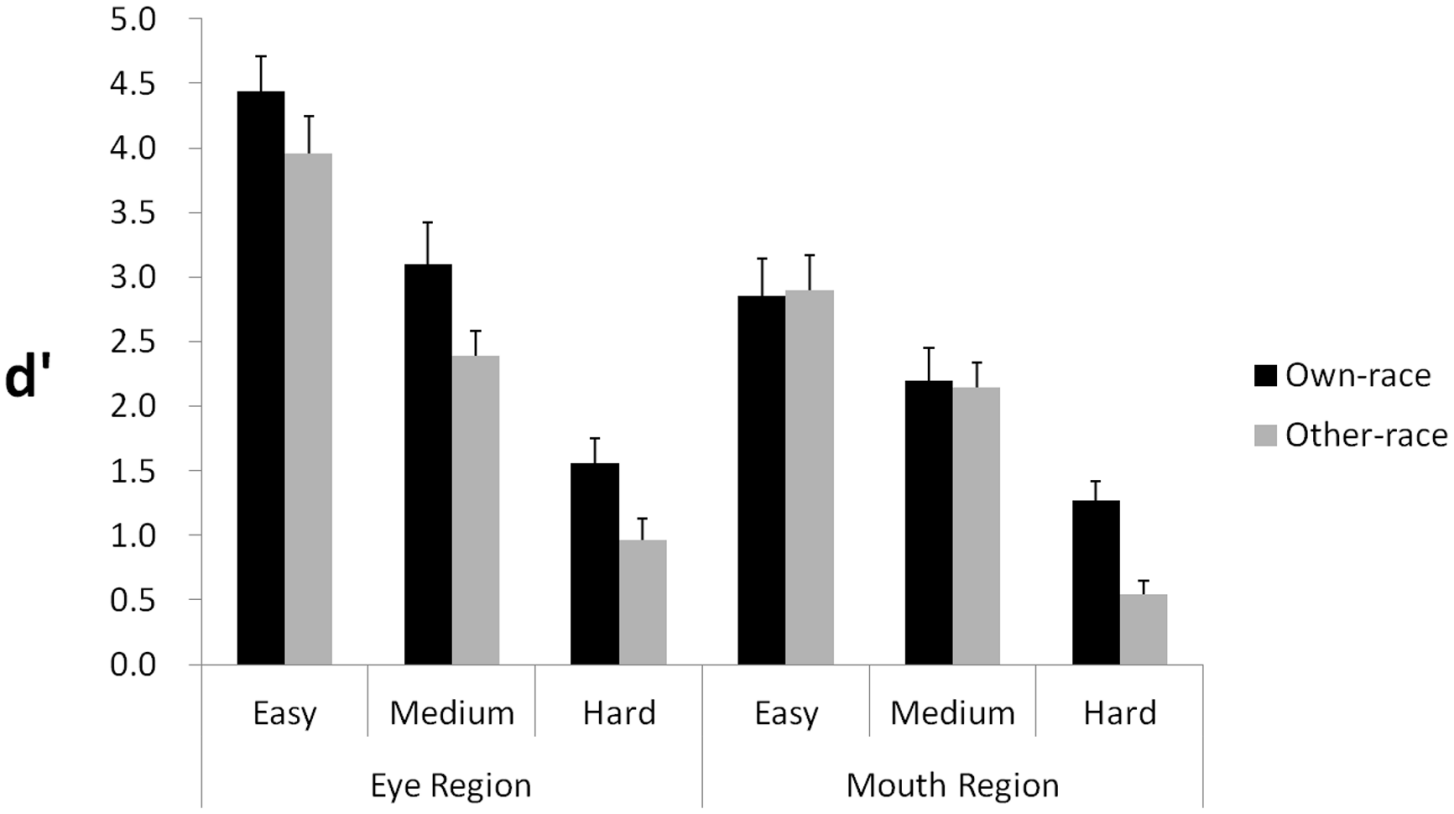
**Experiment 2: manipulating the size of facial features**. The graph shows *d*′ scores for detecting size differences across three levels of change (easy, medium, hard) in the eye and mouth regions, respectively. Error bars represent SEs in each condition.

By using correct “different” responses as Hits, incorrect “different” responses as False Alarms, the equation H = P(“different”—Different) = Φ [(–k + d’)$\sqrt{2}$] + Φ[(-k - d′)/$\sqrt{2}$], and the equation FA = P(“different”—Same) = 2Φ –k/$\sqrt{2}$), *d′*scores were calculated as the dependent measure (Macmillan and Creelman, 2005, p. 197). Results are shown in **Figure 4**.

A 2 (Race: own, other) × 2 (Region: Upper, Lower) × 3 (Level: Easy, Medium, Hard) repeated measures analysis of variance was performed, with all three independent variables as within-subjects factors. The main effect of Race was significant, *F*(1,18) = 44.60, *p* < 0.0001, $\eta_{p}^{2}$ = 0.712, demonstrating an advantage for discriminating own-race size change over other-race size change. The main effect of Region was also significant, *F*(1,18) = 14.60, *p* = 0.001, $\eta_{p}^{2}$ = 0.448, showing that discriminating size change in the upper region was easier than in the lower region. The main effect of level was additionally significant, *F*(2,36) = 144.60, *p* < 0.001, $\eta_{p}^{2}$ = 0.889, indicating that performance varied according to the three levels of discrimination.

With respect to interactions, we found Region interacted with Level, *F*(2,36) = 9.42, *p* = 0.001, $\eta_{p}^{2}$ = 0.344, but Race did not, *F*(2,36) = 2.40, *p* = 0.105, showing that discrimination of size differences in the face images was affected by region of the face. The interaction between Race and Region is marginally significant, *F*(1,18) = 4.24, *p* = 0.054, $\eta_{p}^{2}$ = 0.191. The three-way interaction of Race, Region, and Level was significant, *F*(2,36) = 3.91, *p* = 0.029, $\eta_{p}^{2}$ = 0.178.

To explore the two-way and three-way interactions further, we also performed ANOVAs on the data for the two regions separately. For the upper region, a 2 (Race: Own vs. Other) × 3 (Level: Easy, Medium, Hard) repeated-measures ANOVA was performed. The main effect of Race was significant, *F*(1,18) = 25.06, *p* < 0.001, $\eta_{p}^{2}$ = 0.58, indicating that participants performed better for own-race faces than for other-race faces, thereby providing evidence that eye size differences contribute to the ORE. The main effect of Level was also significant, *F*(2,36) = 121.44, *p* < 0.001, $\eta_{p}^{2}$ = 0.87, indicating that the more eye size difference between two faces, the better participants differentiated them. The interaction of Race and Level was not significant, *F*(2,36) = 0.29, *p* > 0.75.

For the lower region, a 2 (Race: Own vs. Other) × 3 (Level: Easy, Medium, Hard) repeated-measures ANOVA was performed. The main effect of Level was significant, *F*(2,36) = 59.46, *p* < 0.001, $\eta_{p}^{2}$ = 0.768, indicating that the more mouth size difference between two photos, the better participants differentiated them. The main effect of Race was significant, *F*(1,18) = 7.36, *p* = 0.014. The interaction of Race and Level was also significant, *F*(2,36) = 8.41, *p* < 0.001, $\eta_{p}^{2}$ = 0.32, showing that the main effect of Race was due to an own-race advantage observed at the level of small mouth size change.

Collectively, the findings indicate that the ORE was manifested in sensitivity to size change, but was stronger and more stable for changes in the upper region relative to changes in the lower region. In the lower region of a face, participants showed an inconsistent ORE at different levels of mouth size change.

## General Discussion

In the current study, we examined whether participants are differentially sensitive to configural vs. featural changes or differentially sensitive to such changes in the upper vs. lower regions for own- and other-race faces. First, we found that in the upper region, both configural and featural changes in own-race faces were differentiated significantly better than in other-race faces, across multiple change levels. Second, in the lower region, we found that the featural changes resulted in an inconsistent race difference in participant performance, and the configural changes did not result in any ORE. Taken together, these findings provide direct evidence that individuals are selectively more sensitive to changes around the eye region on own-race faces than on other-race faces.

Our findings show that the ORE has region-selectivity during a face discrimination task. Both types of information (i.e., size and spacing) simultaneously contribute to the ORE. Their effects are localized primarily in the upper region, but not in the lower region of a face. These findings demonstrate that the ORE in face information processing is more influenced by the location of the featural and configural changes than by the type of processing (i.e., featural vs. configural). Extending previous findings showing that participants performed better for blurred or scrambled own-race faces than other-race faces (e.g., Schwaninger et al., 2002; Rhodes et al., 2006; Hayward et al., 2008), the current study indicates that the own-race advantage is due to superior processing of featural and configural information in the upper region of the face. In this way, the region-dependency of face processing noted for face perception (e.g., Turati et al., 2002; Peterson and Eckstein, 2012; Quinn et al., 2013; Tanaka et al., 2014b) and face perception deficits (e.g., Caldara et al., 2005; Bukach et al., 2008; Wolf et al., 2008) has been extended to explain the difference between own- and other-race face processing, suggesting that the location of information in a face should be considered as a key factor in investigating and theorizing about face perception and face processing expertise.

Analogously, judgments of featural and configural variations are similarly affected by the face inversion effect, once processing difficulty or participant expectations for variation-change are equated for the two types of information (Riesenhuber et al., 2004; Sekuler et al., 2004; Yovel and Kanwisher, 2005; McKone and Yovel, 2009). Particularly, Tanaka et al. (2014a) demonstrated that both featural and configural information processing in the eye region were preserved, but both featural and configural information processing were impaired in the mouth region when participants observed an inverted face. To understand these findings and our current results in an integrated way, we would suggest that participants have an *expertise area* (i.e., the eye region), where the processing resolution is higher for (1) information inside relative to information outside, so that eye region processing is preserved, but mouth region processing is impaired during face inversion, and (2) own-race face processing relative to other-race face processing, so that an own-race advantage across information type (i.e., both configural and featural) is shown consistently in the eye region, but not in the mouth region.

It is still an open question as to whether the ORE is sensitive to changes around the nose, relative to changes around the mouth and eyes. Future studies could manipulate not only the eyes and mouth, but also the nose as an isolated feature. We would speculate that the nose area plays a different role in Caucasian versus Asian participants when they respond to Caucasian and Asian faces. This speculation is based on recent eye-tracking data showing that Asian participants spend a significantly greater proportion of fixation time on the nose of Asian faces than on the nose of Caucasian faces (e.g., Blais et al., 2008; Caldara et al., 2010; Kelly et al., 2010; Fu et al., 2012). Also, since the current study tested only Asian participants, it will become important to test both Asian and Caucasian participants to firmly rule out the influence of low-level visual properties of the stimuli (Vizioli et al., 2010a,b) and determine whether the effect of location may extend across multiple races of perceivers. Given that the effects of spacing and size on the key facial features were investigated separately in the current study, the question of whether these effects might interact also remains open. Future studies might therefore vary spacing and size at the same time to test the effect of the interaction between these two factors.

In summary, we examined the sensitivity of observers to configural and featural changes in the upper and lower regions of own- and other-race faces. Our data reveal that the ORE is more pronounced when featural and configural changes are in the upper region than in the lower region. These findings indicate that information from the upper vs. lower region of the face contributes differentially to the ORE in face processing, and that processing of face race is influenced more by information location (i.e., upper vs. lower) than by information type (i.e., configural vs. featural).

## Conflict of Interest Statement

The authors declare that the research was conducted in the absence of any commercial or financial relationships that could be construed as a potential conflict of interest.
