# Erratum: An other-race effect for configural and featural processing of faces: upper and lower face regions play different roles

**DOI:** 10.3389/fpsyg.2015.01517

**Published:** 2015-09-29

**Authors:** Frontiers Production Office

**Affiliations:** Frontiers Production Office, FrontiersLausanne, Switzerland

**Keywords:** face processing, face recognition, other-race effect, Face Dimensions Test, configural information, featural information, upper vs. lower face

Reason for Erratum:

Due to a typesetting error, the reviewer Caroline Blais was inadvertently removed from the final published article. The publisher apologizes for this mistake.

This error does not change the scientific conclusions of the article in any way.

Original article has been updated

